# In vitro propagation method for production of morphologically and genetically stable plants of different strawberry cultivars

**DOI:** 10.1186/s13007-019-0421-0

**Published:** 2019-04-13

**Authors:** Aung Htay Naing, Si Hyun Kim, Mi Young Chung, Soon Ki Park, Chang Kil Kim

**Affiliations:** 1 Department of Horticultural Science, Kyungpook National University, Daegu, 4165122 South Korea; 2 School of Applied Biosciences, Kyungpook National University, Daegu, South Korea; 3 Department of Agricultural Education, Suncheon National University, Suncheon, South Korea

**Keywords:** Field performance, Fruit quality, Genetic variation, Kinetin, Meristem

## Abstract

**Background:**

As strawberries are susceptible to somaclonal variation when propagated by tissue culture techniques, it is challenging to obtain the true-to-type plants necessary for continuous production of fruits of stable quality. Therefore, we aimed to develop an in vitro propagation method for the production of true-to-type plants of five different strawberry cultivars from meristems cultured in media containing different concentrations of kinetin (Kn).

**Results:**

For all the cultivars, shoot induction was successful only in the meristems cultured in the medium without Kn and the medium containing 0.5 mg L^−1^ Kn. The shoots obtained from explants cultured in media supplemented with 0.5 mg L^−1^ Kn exhibited better plant growth parameters than those cultured in media without Kn and were genetically stable when compared with conventionally propagated plants for all the cultivars. Vegetative and sexual characters and fruit quality attributes observed in the plants derived from meristems cultured on 0.5 mg L^−1^ Kn and the conventionally propagated plants were not significantly different when grown for three continuous growing seasons under greenhouse conditions.

**Conclusion:**

The culture of meristems in the medium containing 0.5 mg L^−1^ Kn is suitable for the efficient propagation of true-to-type plants of different strawberry cultivars and continuous production of fruits with stable quality. Hence, we expect that the method presented in this study will be helpful for the commercial production of true-to-type plants generated in vitro for other strawberry cultivars.

**Electronic supplementary material:**

The online version of this article (10.1186/s13007-019-0421-0) contains supplementary material, which is available to authorized users.

## Background

Strawberry is one of the most consumed fruits owing to its fragrance, taste, antioxidant capacity resulting from high levels of anthocyanins, and other nutritional properties [1]. Cultivation of the strawberry has greatly increased, and this fruit has become one of the most cultivated fruit crops worldwide. However, the provision of a sufficient quantity of true-to-type plants for commercial production is challenging. Conventional propagation by runner segments limits the number of propagules, and the obtained propagules are susceptible to plant diseases such as those caused by fungi [2], *Strawberry mottle virus* (SMoV), and *Strawberry mild* yellow edge virus (SMYEV), which gravely threaten strawberry production [3]. In vitro propagation techniques using different explant types, such as node cultures [4] and leaf, sepal, and petiole explants [5–7], have been widely implemented for efficient strawberry production. Unfortunately, strawberry plants obtained through tissue culture are pliable to genetic variations in the field, exhibited by changes in growth habits such as greater branching, vigorous vegetative growth, and greater number of crowns and runners when compared with conventionally propagated plants [8–10]. Meristem culture has been used for the production of disease-free strawberry plants from infected plants [11, 12]. Recently, Munri et al. [13] conducted successful in vitro propagation of different strawberry cultivars from meristem cultures. However, meristem-derived plants also show morphological changes under field conditions [14–16]. Occurrence of such variation in tissue culture-derived plants seriously limits the utility of the in vitro propagation system.

Previous studies reported that the application of low concentrations of cytokinins [16, 17], a reduced number of subcultures during the proliferation stage [18, 19], and the choice of genotypes [20, 21] are critical factors that should be considered when obtaining true-to-type plants. Hence, it is necessary to develop an efficient protocol that can effectively produce genetically stable, virus-free shoots of different cultivars using the meristems.

In the present study, in vitro shoot regeneration of five different cultivars was investigated by culturing the meristems in media containing different concentrations of kinetin (Kn). Genetic stability of meristem-derived and conventionally propagated plants was assessed using a molecular marker (random amplification of polymorphic DNA, RAPD) and flow cytometry. In addition, vegetative traits, sexual characters, and fruit attributes of the meristem-derived and conventionally propagated plants were compared during three continuous growing seasons under greenhouse conditions.

## Materials and methods

### Plant materials

**Fig. 1:**
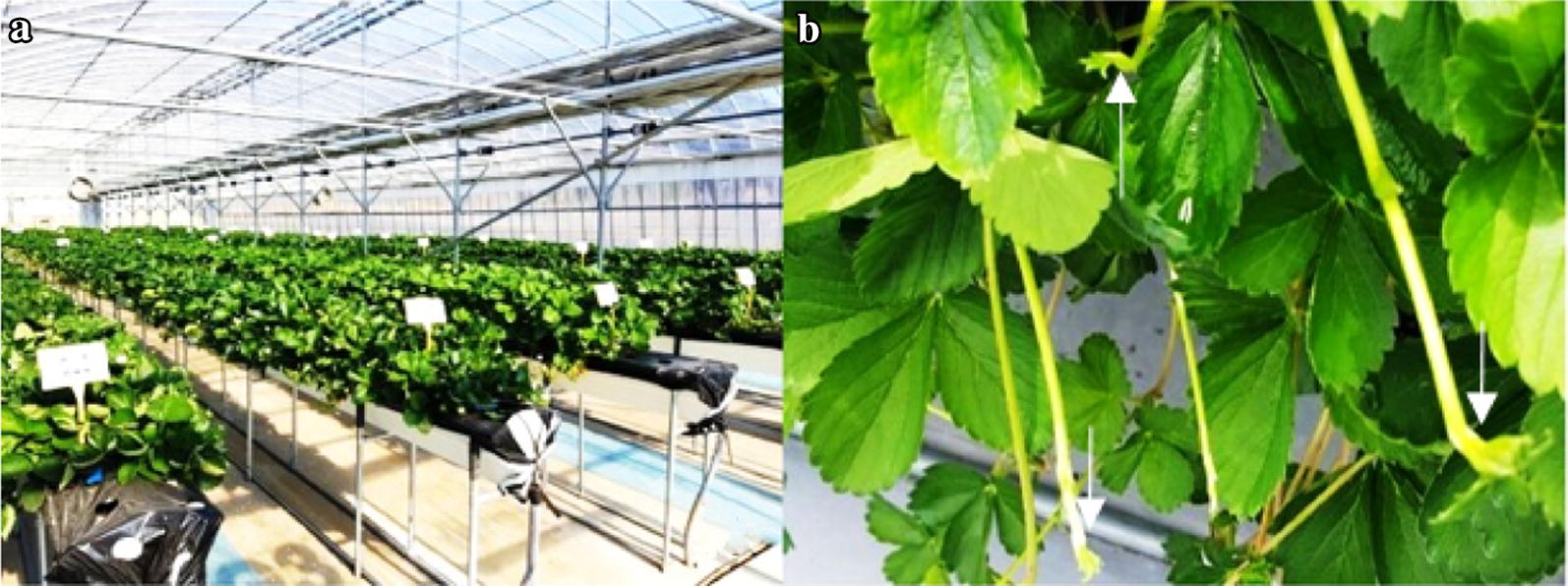
Mother plants grown in the greenhouse (**a**) and runner tips with one leaf primordia (**b**) used as plant materials. Arrows indicate apical meristems

During late September to early November, about 10 cm long runner tips with one leaf primordium were collected from five strawberry cultivars, Santa, Fanta, Berrystar, Honeybell, and Okhyang, grown as donor plants in a greenhouse at the Seongju Fruits and Vegetable Experimental Station, Seongju, Korea (Fig. 1a, b). The collected runners were thoroughly washed under tap water, and 2 cm long runner tips were excised for aseptic sterilization. The aseptic sterilization was done by dipping the runner tips in sterile distilled water containing 1% sodium hypochlorite (NaOCl) and a drop of Tween 20 for 10 min. The sterilized runners were then rinsed with sterile distilled water at least three times and used as explants for meristem culture.

### In vitro shoot regeneration using meristem culture

Meristems (0.3–0.5 mm) were dissected using a stereoscopic microscope from the sterilized runners of the five cultivars under aseptic conditions. The meristems were immediately cultured in test tubes containing Murashige and Skoog (MS) [22] basal medium and different concentrations of Kn (0 [control], 0.5, 2.0, 3.0, and 4.0 mg L^−1^), 3% sucrose, and 0.8% agar, and the pH of 5.8. The explants were incubated in a culture room at 25 °C under a photoperiod of 16 h. After 8 weeks of culture, explants (from the 0.5 mg L^−1^ Kn and control treatments) producing crowns with shoots were subcultured without separating individual shoots (in order to avoid damaging shoots and because separation would have been prohibitively time-intensive) in a culture bottle containing 0.75 mg L^−1^ indole-3-butyric acid (IBA), 3% sucrose, and 0.8% agar, and a pH of 5.8, for rooting and plant growth. After 6 weeks of culture, the average number of roots, shoots, and leaves per explant was evaluated for each cultivar. Each treatment contained thirty explants (test tubes), and there were three replications per treatment.

### Acclimatization of meristem-derived regenerated plants

To reduce transplanting shock, rooted shoots of the subcultured explants were not separated before transplanting them to pots with soil. The rooted shoots were transplanted into plastic trays filled with peat-based soil and acclimatized in a growth chamber for 3 weeks under relative humidity of 100% the first week of acclimatization and 70% for the next 2 weeks. After the acclimatization period, the number of surviving shoots per explant was recorded. Each treatment contained 30 explants (test tubes), and there were three replications per treatment. For the evaluation of genetic variation, individual rooted shoots were separated from the explants (only from those derived from cultures on 0.5 mg L^−1^ Kn) and transferred into plastic pots containing peat-based soil. The pots were placed in a greenhouse at 22 °C and the vegetative characters, sexual characters, and fruit quality attributes of the plants were evaluated.

### Analysis of ploidy variation by flow cytometry

Conventionally propagated runners from the five strawberry cultivars, grown in the greenhouse as donor plants and provided by the Seongju Fruits and Vegetable Experimental Station, Seongju, Korea, were used as control plants. The conventionally propagated plants and meristem-derived plants grown in the greenhouse were randomly selected for analysis of ploidy levels. The ploidy levels were determined by flow cytometry as described by Naing et al. [23]. Briefly, about 20 mg of leaf tissue per plant was chopped with a razor blade in a plastic petri dish containing nuclei extraction buffer (Partec GmbH, Münster, Germany), and the obtained suspensions were filtered through a nylon mesh (50 lm). Subsequently, the filtered solution was mixed with 1.6 mL of staining buffer (Partec GmbH, Germany), followed by propidium iodide. The ploidy level from each sample was measured using a flow cytometer (Partec GmbH, Germany). The conventionally propagated plants were used as an octaploid reference. Three different biological samples were prepared for both conventionally propagated and tissue culture-derived plants.

### Detection of genetic variation by RAPD

Total genomic DNA was isolated from the leaves of both the conventionally propagated plants and meristem-derived plants using a HiYield genomic DNA mini Kit (Real Biotech Corporation, Taipei, Taiwan). RAPD amplification was performed as described by Biswas et al. [16]. The primers and PCR conditions for the amplification are given in Additional file 1: Table S1. The reaction products were analyzed via electrophoresis on a 2% (w/v) agarose gel stained with ethidium bromide and photographed under UV light exposure. RAPD analysis using each primer was repeated for all the cultivars at least three times to verify the banding pattern of the studied DNA samples.

### Evaluation of vegetative characters, sexual characters, and fruit quality attributes under greenhouse conditions

Conventionally propagated plants and meristem-derived plants (obtained from explants cultured on the media containing 0.5 mg L^−1^ Kn) of the same size were planted at the same time in separate beds located in the greenhouse. The experimental design was a completely randomized block with three replications (three beds each). Each replication contained 15 plants. After 13 weeks of cultivation, canopy size, number of leaves and shoots, and number of flowers per plant were recorded and compared. In addition, mature ripe fruits with a well-developed red color were harvested from both groups of plants, and fruit fresh weight, fruit width, fruit length, firmness, and total sugar content per fruit were measured. In the next growing season, the same data were collected for daughter plants of the meristem-derived and conventionally propagated plants as described above. The experiment was repeated in three consecutive growing seasons.

### Detection of flesh firmness and total sugar content

Once the fruits of all cultivars were harvested, fruits of uniform size were selected from each cultivar and each individual fruit was cut in half. Both halves of each fruit were analyzed for flesh firmness and total sugar content. Flesh firmness was determined using a Rheo-meter (Compac-100II; Sun Scientific Co., Tokyo, Japan), and total sugar content was measured using a Digital Refractometer GMK-703AC (G-won hightech, Korea), as described by Lee et al. [24]. The firmness and sugar content measurements were taken from the stem end of fruits, where the fruits were broadest. Each measurement was conducted using 10 different individual fruits of each cultivar of conventionally propagated and tissue culture-derived plants in each of the three growing seasons. This experiment was repeated three times (10 fruits × 5 cultivars × 2 plant types × 3 growing seasons × 3 replications of the experiment = 900 total fruit evaluated per measurement type).

### Statistical analysis

Data were analyzed using SPSS v. 11.09 (IBM Corporation, Armonk, NY, USA), and are presented as the mean of three replicates. Means were analyzed using *t*–tests with a significance level of *P* < 0.05.

## Results

### Effects of kinetin on in vitro shoot regeneration from meristem

**Fig. 2:**
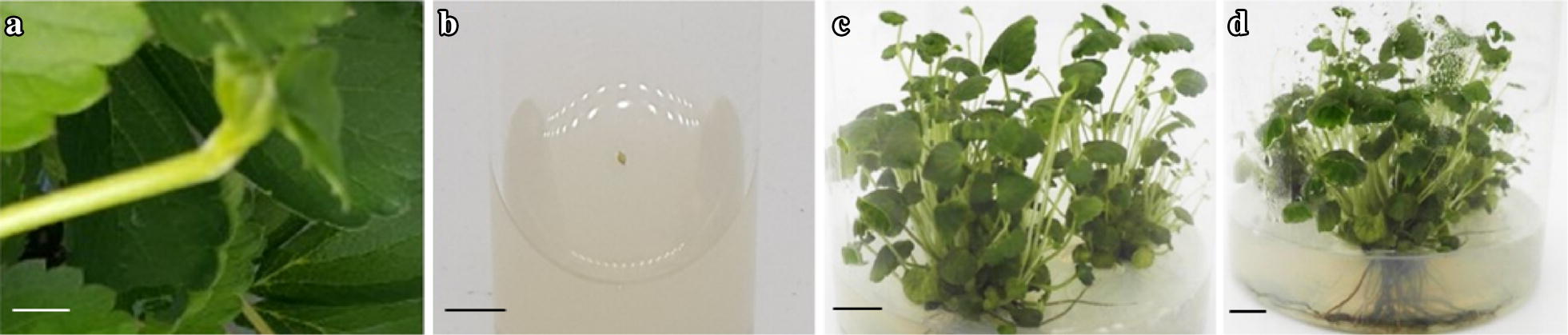
In vitro regeneration of shoots from meristems: **a** runner tip used as explant source; **b** culture of the meristem excised from the runner tips on the medium containing Kn 0.5 mg L^−1^; **c** induction of shoots from the meristem after 8 weeks of culture; **d** rooting of the meristem-derived plants on the rooting media containing indole-3-butyric acid after 6 weeks of culture. Size bars for figures (**a**–**d**) indicate 1.0, 0.5, 1.0, and 1.0 cm, respectively

**Fig. 3:**
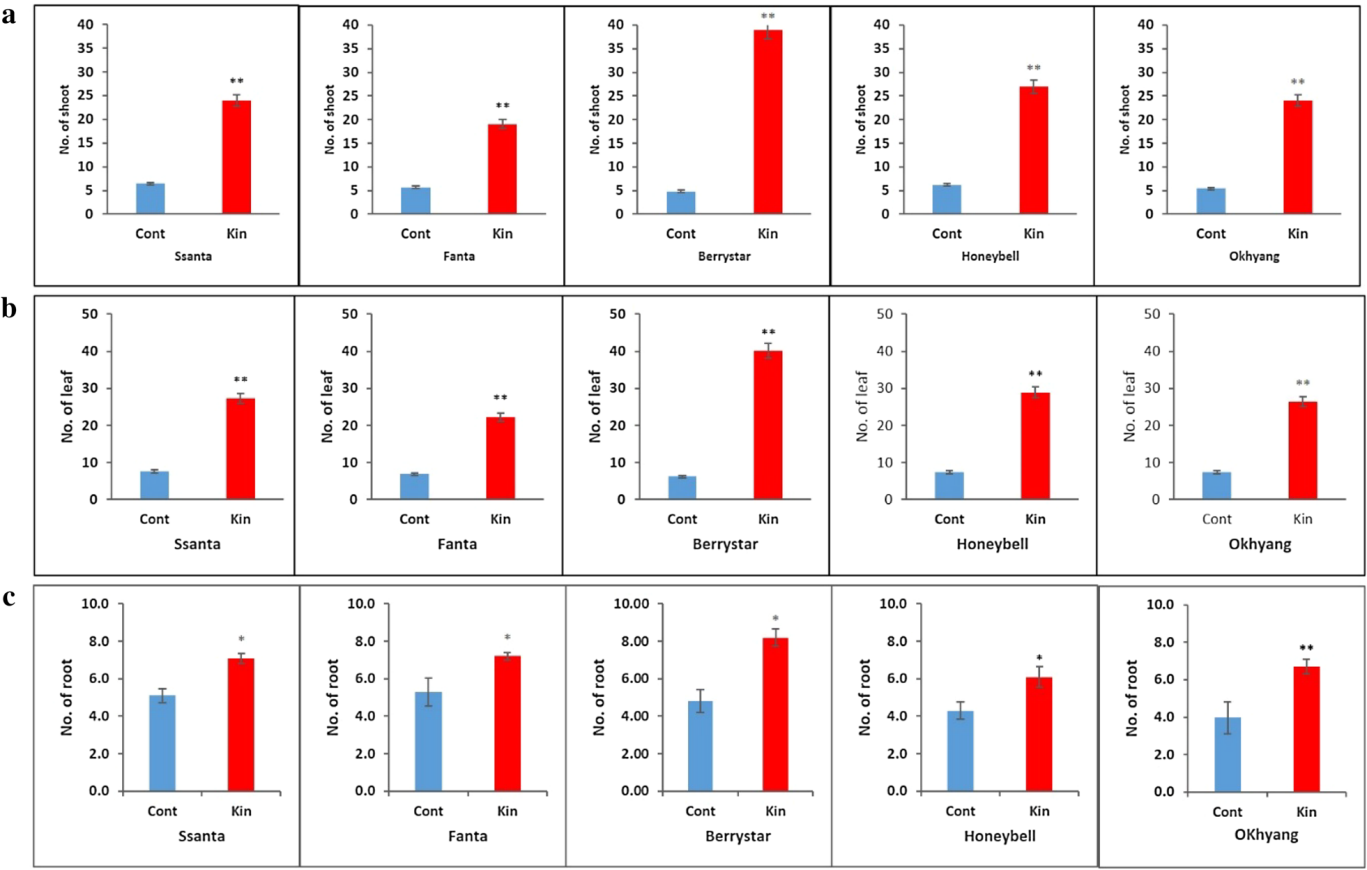
Effect of kinetin on in vitro regeneration of shoots from the meristem of different strawberry cultivars after 14 weeks of culture: **a** number of shoots; **b** number of leaves; **c** number of roots per shoot. Data represent the mean of three replicates, and the bar indicates the standard deviation. Means with asterisk(s) are statistically significant (*t*-test, *P < 0.05)

The response of the meristems of the five strawberry cultivars cultured on MS media containing different concentrations of Kn varied with Kn concentration and the cultivar type. For all the cultivars, callus initiation commenced after 3 weeks of culture. However, the initiated calluses did not convert into shoots in the media containing 2.0, 3.0, and 4.0 mg L^−1^ Kn and gradually died after 8 weeks of culture (data not shown). Shoot induction was only observed in explants cultured on the medium containing 0.5 mg L^−1^ Kn and in the control. When these explants producing shoots were sub-cultured on the medium containing 0.75 mg L^−1^ IBA, shoot growth improved and roots developed after 6 weeks of culture (Fig. 2a–d). For all the cultivars, percentage shoot regeneration, the number of shoots, leaves, and roots per explant derived from explants cultured on the medium containing 0.5 mg L^−1^ Kn was significantly higher than that derived from the control (Fig. 3a–c, Table 1), indicating that the addition of 0.5 mg L^−1^ Kn provides optimal conditions for successful in vitro propagation of strawberry cultivars through meristem culture.

**Table 1:** Effect of kinetin on in vitro shoot regeneration percentages from the meristem of different strawberry cultivars after 14 weeks of culture

Treatment (mg L^−1^)	Cultivars				
	Santa	Fanta	Berrystar	Honeybell	Okhyang
	Shoot regeneration (%)	Shoot regeneration (%)	Shoot regeneration (%)	Shoot regeneration (%)	Shoot regeneration (%)
Control (Kn 0)	70	68	75	64	69
Kn 0.5	88*	88*	90*	83*	87*

Data represent the mean of three replicates, and the means with asterisk(s) are statistically significant (*t*-test, *P <0.05)

### Acclimatization of regenerated plants

**Fig. 4:**
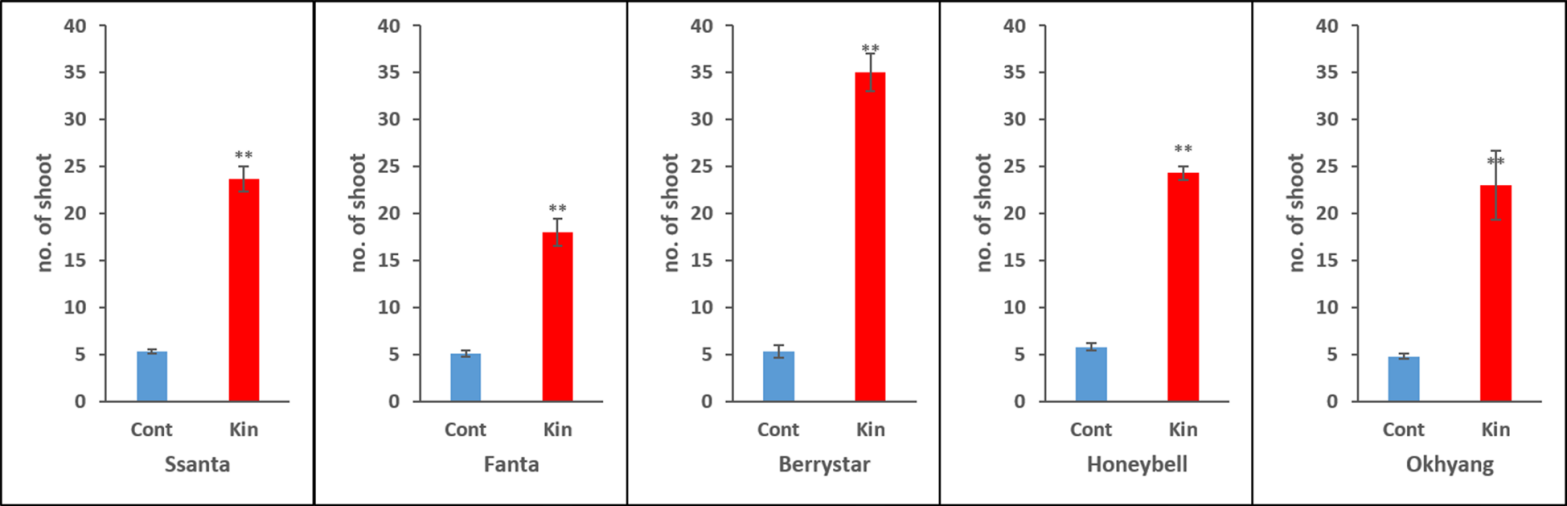
Number of surviving plants per explant of different strawberry cultivars after acclimatization. Data represent the mean of three replicates, and the bar indicates the standard deviation. Means with asterisk(s) are statistically significant (*t*-test, *P < 0.05)

**Fig. 5:**
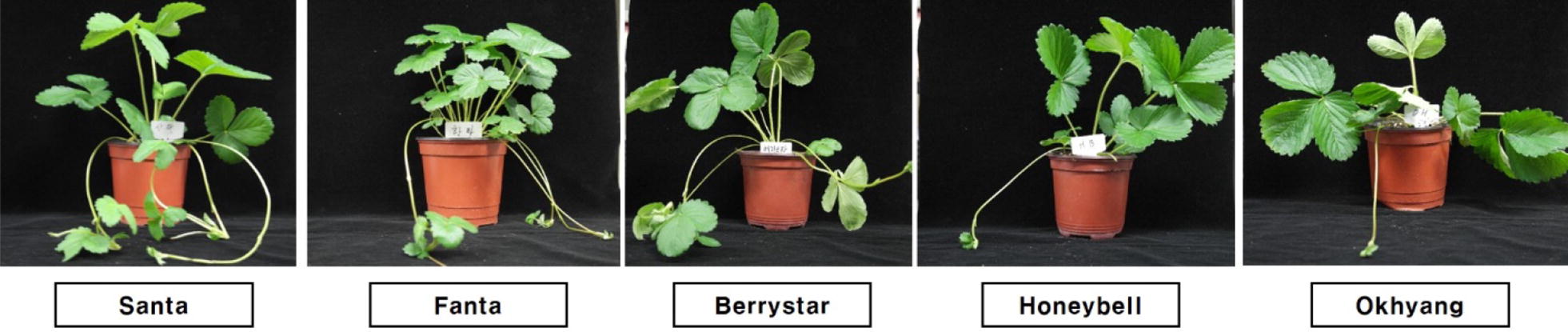
Meristem-derived plants (obtained from explants cultured on medium supplemented with 0.5 mg L^−1^ kinetin) of different strawberry cultivars cultivated in the greenhouse

To assess the sustainability of rooted shoots derived from control explants and those cultured on media supplemented with 0.5 mg L^−1^ Kn, the shoots, while still attached to explants, were acclimatized in the growth chamber for 3 weeks. Most of the shoots survived during the acclimatization period, and, as expected, the number of surviving shoots per explant derived from those cultured on 0.5 mg L^−1^ Kn-enriched media was significantly higher than that in explants derived from control plants for all the cultivars (Fig. 4). Moreover, the plant growth of the former plants was superior to that of the control plants, and these plants thrived when grown in pots in a greenhouse (Fig. 5).

### Detection of ploidy levels by flow cytometry

**Fig. 6:**
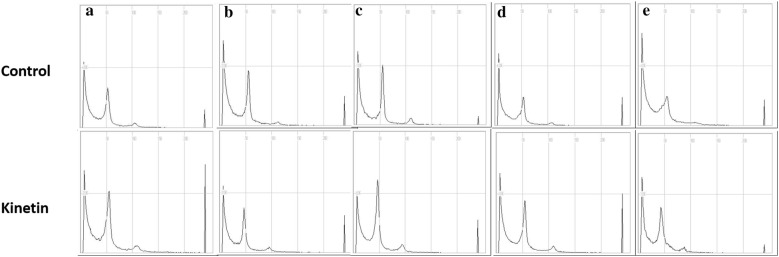
Comparison of the ploidy levels between meristem-derived plants (Kinetin) and conventionally propagated plants (control) of different strawberry cultivars: **a** Santa; **b** Fanta; **c** Berrystar; **d** Honeybell; **e** Okhyang

The ploidy level of the conventionally propagated plants and meristem-derived plants cultured on medium containing 0.5 mg L^−1^ Kn was determined by flow cytometry. The typical flow cytometry profiles indicated no variation in the ploidy levels between the two groups of plants grown in the greenhouse for all the cultivars (Fig. 6a–e).

### RAPD analysis

**Fig. 7:**
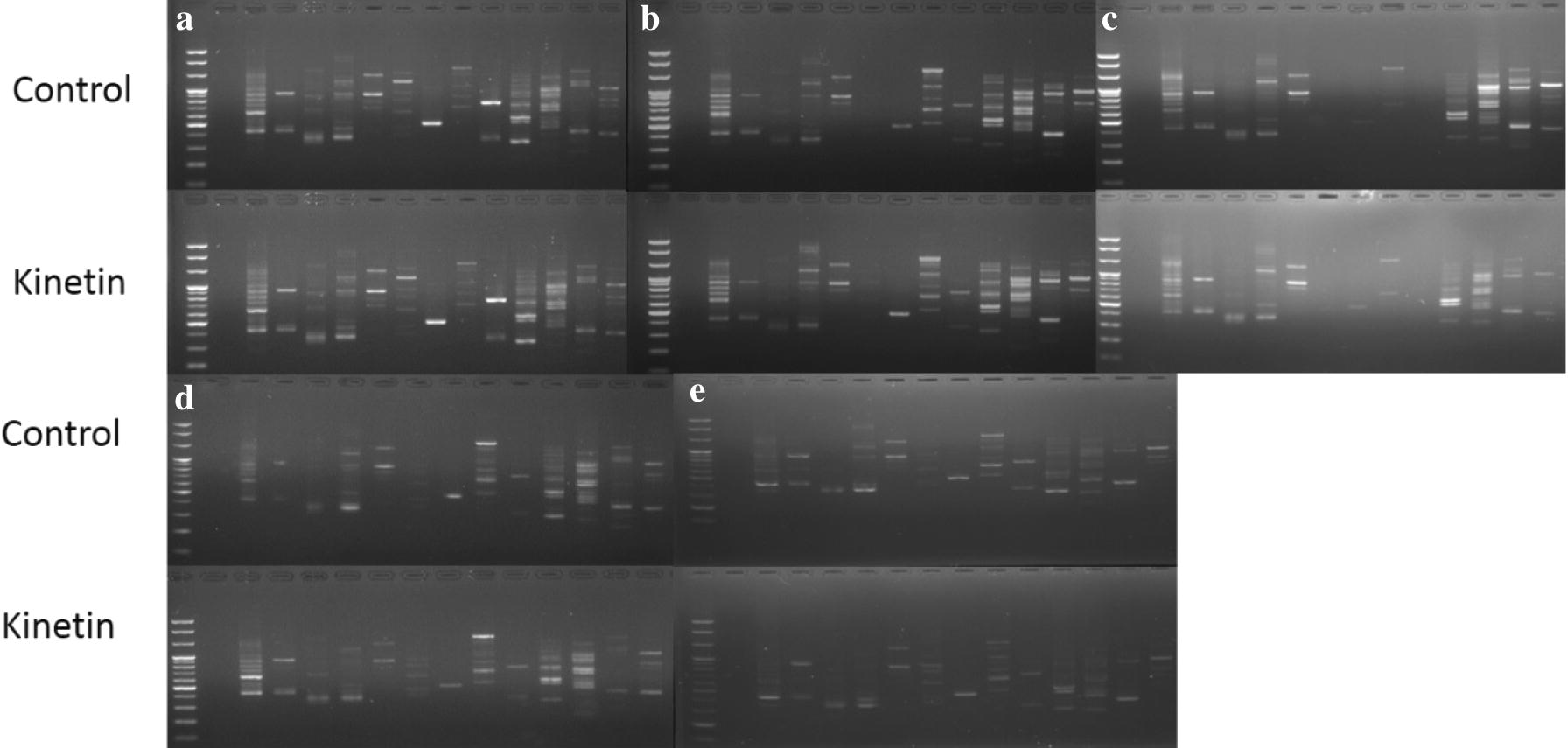
Detection of somaclonal variation between meristem-derived plants (Kinetin) and conventionally propagated plants (control) of different strawberry cultivars using RAPD markers: **a** Santa, **b** Fanta, **c** Berrystar, **d** Honeybell, **e** Okhyang

Most of the 14 random primers used in the RAPD analysis successfully produced scorable bands for both the conventionally propagated and meristem-derived plants, but the RAPD banding patterns were slightly different among the tested cultivars (Fig. 7a–e). As expected, the banding patterns observed in meristem-derived plants were similar to those of the conventionally propagated plants for all the cultivars.

### Evaluation of vegetative characters, sexual characters, fresh fruit firmness, and total sugar content

**Fig. 8:**
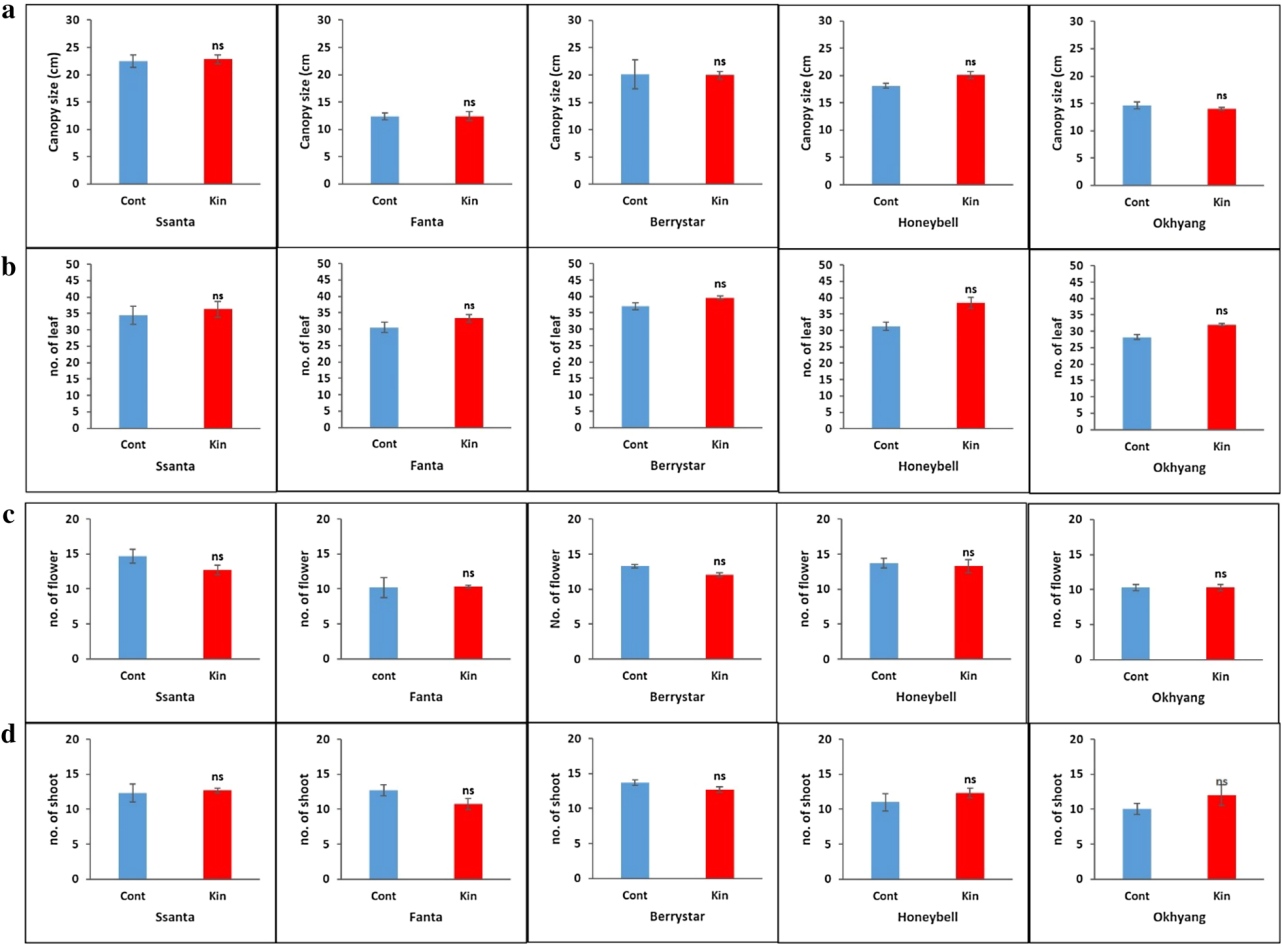
Comparison of vegetative and sexual traits between meristem-derived plants (Kin) and conventionally propagated plants (Cont) of different strawberry cultivars: **a** canopy size; **b** number of leaves; **c** number of flowers; **d** number of shoots. Data represent the mean of three growing seasons, and the bar indicates the standard deviation. Means with asterisk(s) are statistically significant (*t*-test, *P < 0.05)

**Fig. 9:**
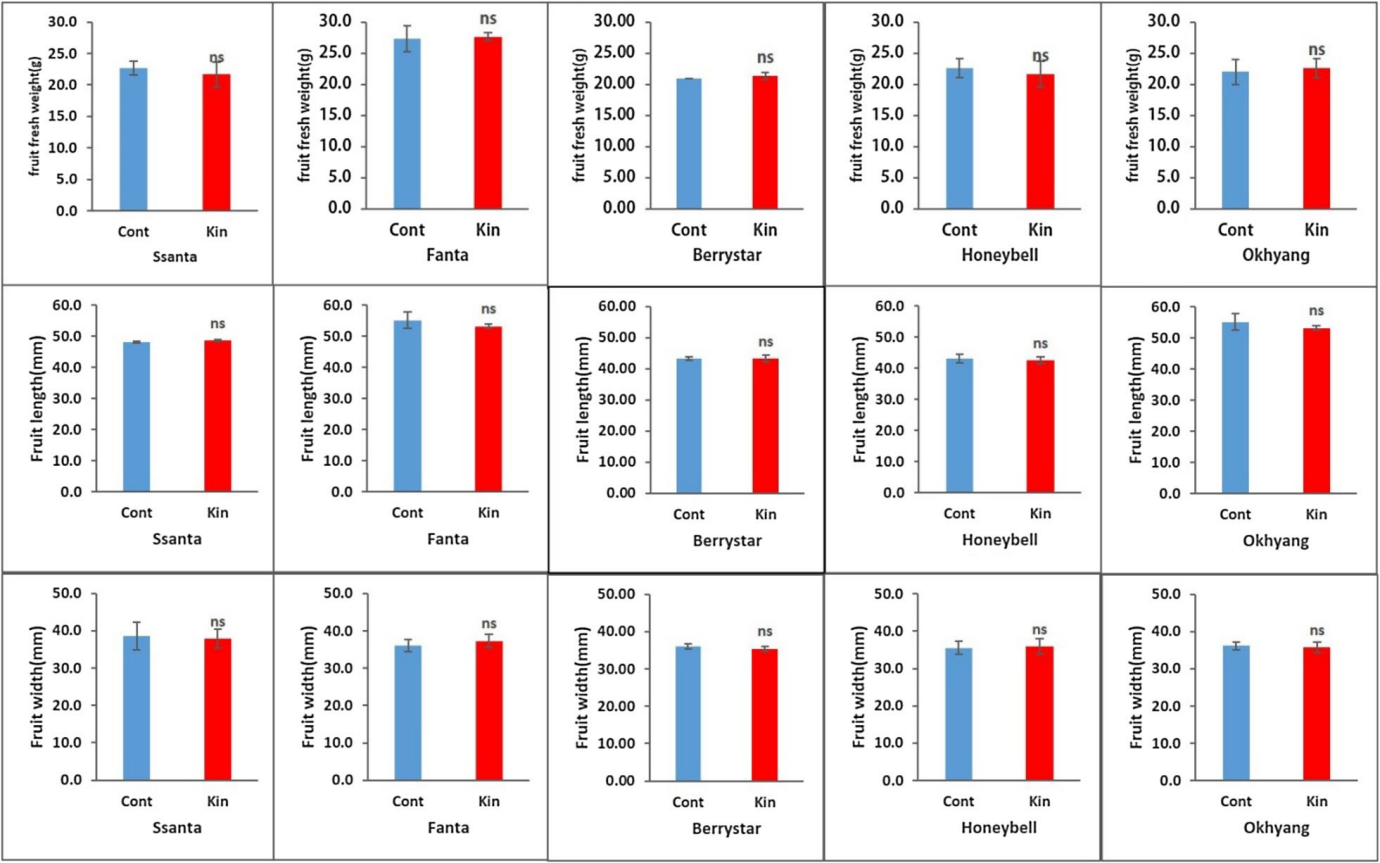
Comparison of fruit characters between meristem-derived plants (Kin) and conventionally propagated plants (Cont) of different strawberry cultivars: **a** fruit fresh weight; **b** fruit length; **c** fruit width. Data represent the mean of three growing seasons, and the bar indicates the standard deviation. Means with asterisk(s) are statistically significant (*t*-test, *P < 0.05)

**Fig. 10:**
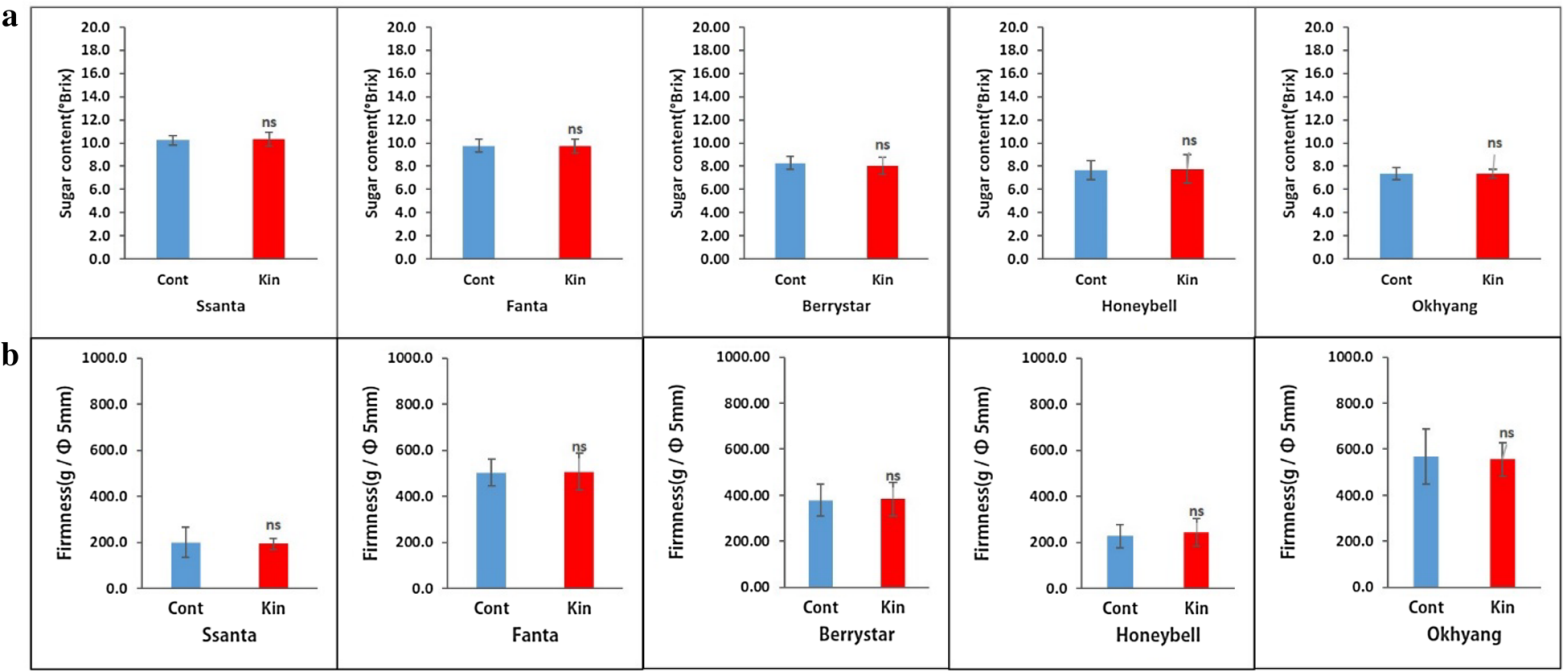
Comparison of total sugar content (**a**) and firmness (**b**) per fruit between meristem-derived plants (Kin) and conventionally propagated plants (Cont) of different strawberry cultivars. Data represent the mean of three growing seasons, and the bar indicates the standard deviation. Means with asterisk(s) are statistically significant (*t*-test, *P < 0.05)

For all the cultivars, the conventionally propagated plants and meristem-derived plants (regenerated from explants cultivated on media supplemented with 0.5 mg L^−1^ Kn) were grown in the greenhouse until fruit ripening and maturity. During the growing period, the canopy size, number of leaves and shoots, and number of flowers per plant, as well as the fruit fresh weight, fruit width, fruit length, firmness, and total sugar content per fruit were recorded for three continuous growing season, and the data were averaged and compared between the two groups of plants. The vegetative traits, such as the canopy size and the number of leaves and shoots, and the number of flowers per plant, were not significantly different between the two groups of plants for all the cultivars (Fig. 8a–d). Similarly, the fruit fresh weight, width, and length (Fig. 9a–c) and fruit firmness and total sugar content (Fig. 10a, b) were not significantly different between the two plant groups for all the cultivars.

## Discussion

In vitro propagation systems using different types of explants and plant growth regulators have been developed for reproduction of strawberry [6, 7, 13, 16, 25, 26]. However, there have been no reports describing propagation systems for Santa, Fanta, Berrystar, Honeybell, and Okhyang, the cultivars commercially grown in Korea. Genetic variation in tissue culture-derived plants can be induced by the application of higher concentrations of cytokinins, frequent subculturing during proliferation, and the choice of genotype [16, 17, 19–21]. Hence, by implementing these findings, we established an efficient in vitro propagation protocol for reproduction of these five cultivars (Santa, Fanta, Berrystar, Honeybell, and Okhyang) from the meristem to obtain genetically stable and virus-free regenerated plants, the properties preferred by commercial growers. Greenhouse performance of the meristem-derived plants was compared with that of plants conventionally propagated from donor plants grown in a greenhouse, and the stability of fruit quality was evaluated for three continuous growing seasons.

In all the cultivars, the shoot growth was induced from the meristem cultured on both the medium without Kn (control) and that with low concentration of Kn (0.5 mg L^−1^), although the induction was stronger in the latter. Surprisingly, no shoot induction was observed in the media containing more than 0.5 mg L^−1^ (data not shown), which may be due to Kn toxicity to plant cells and tissues. After transferring the explants to the rooting and plant growth medium, the number of shoots that developed per explant was significantly higher in plants cultured on the medium containing Kn 0.5 mg L^−1^ than in the control. In addition, the shoots induced by 0.5 mg L^−1^ Kn were likely to be healthier and produced more leaves and roots than those derived from explants grown in the Kn-free medium. This improved growth performance may be explained by the promoting effect of Kn on cell division, leading to higher number of shoots, leaves, and roots. A greater number of surviving shoots and superior plant growth were also observed in explants derived from cultures on the medium with 0.5 mg L^−1^ Kn during acclimation, which may be attributed to their primarily healthier shoots as compared to those derived from the control. Furthermore, these Kn-derived plants thrived in the greenhouse conditions and were genetically stable in comparison with conventionally propagated plants, as indicated by flow cytometry and RAPD markers.

Genetic variation was assessed by RAPD markers in in vitro-regenerated plants derived from different explant sources in strawberry [15, 16, 21, 27, 28]. Nehra et al. [28] observed a somaclonal variation in meristems cultured in a medium containing 5 μM benzyladenine (BA), while Biswas et al. [16] reported that the application of high concentrations of 6-benzylaminopurine (BAP) induced genetic variation in meristem-derived plants. The combination of 4.4 mg L^−1^ BA and 4.5 mg L^−1^ 2,4-dichlorophenoxyacetic acid (2, 4-D) similarly induced genetic variation in callus-derived plants [21]. Moreover, Popescu et al. [21] reported genetic variation in leaf-derived plants obtained by culture on media containing 4.4 mg L^−1^ BAP. Because of the previous reports of genetic instability in in vitro generated strawberry plants, we assessed whether ploidy variation would occur in the meristem-derived plants using flow cytometry, followed by analysis of genetic variation using RAPD markers. We detected no ploidy or genetic variation between the meristem-derived plants (cultured on media supplemented with 0.5 mg L^−1^ Kn) and conventionally propagated plants for all the cultivars. This discrepancy between previous studies and the present study could be due to differences in the presence and concentrations of plant growth regulators. To the best of our knowledge, application of Kn in strawberry meristem culture is relatively less to date. The optimal concentration of Kn that induced shoot initiation used in the present study was not too high when compared with the levels of other plant growth regulators used in previous studies. Therefore, lower concentrations of Kn than the typical concentrations of BA, BAP, and 2, 4-D are more appropriate for commercial production of genetically stable strawberry plants from meristems.

In comparison with conventionally propagated runner plants, meristem-derived plants with somaclonal variations exhibited changes in growth habit, runner production, and other vegetative and sexual characters [14–16]. Nehra et al. [28] reported that meristem-derived plants of the strawberry cultivar Redcoat produced more flowers and fruits than conventionally propagated plants. In the present study, the meristem-derived plants and conventionally propagated plants cultivated in the greenhouse for three growing seasons did not differ in canopy size, number of leaves and shoots, number of flowers per plant, and fruit quality attributes such as single fruit fresh weight, fruit width, fruit length, fruit firmness, and total sugar content. These results were attributed to the genetic stability of the meristem-derived and conventionally grown plants. Taken together, the results presented herein indicate that Kn at a concentration of 0.5 mg L^−1^ efficiently induced the growth of genetically stable plants from the meristem. These plants were morphologically stable and virus-free when propagated and grown under greenhouse conditions for three continuous growing seasons. The method used in this study can therefore efficiently induce genetically and morphologically stable shoots from meristems, a major difference compared with the results of previous studies. We expect that this novel method can ensure a continuous supply of strawberry fruits with stable quality to markets. Moreover, from the economical point of view, this approach eradicates virus diseases that cause serious yield losses in every growing season.

## Conclusion

We established an in vitro propagation method for five strawberry cultivars by culturing the meristem in MS medium containing different concentrations of Kn. The concentration of 0.5 mg L^−1^ Kn produced the most optimal shoot induction and plant growth parameters. The resulting meristem-derived plants were genetically stable in comparison with conventionally propagated plants, and the vegetative growth and fruit quality attributes of the meristem-derived and conventionally propagated plants were similar when cultivated in a greenhouse for three continuous growing seasons. Therefore, we expect that the in vitro propagation method implementing 0.5 mg L^−1^ Kn will be applicable in commercial production of virus-free strawberry plants with stable fruit quality in other strawberry cultivars.

## Additional file

**Additional file 1.** List of primers used for RAPD analysis.
