# Supplementary material for: In vitro propagation method for production of morphologically and genetically stable plants of different strawberry cultivars
Source: Plant Methods. 2019 Apr 13;15:36. doi: 10.1186/s13007-019-0421-0 (PMC6461810; doi:10.1186/s13007-019-0421-0)
Supplement: Supplementary file 1 — Additional file 1. List of primers used for RAPD analysis. [file 13007_2019_421_MOESM1_ESM.docx]

| **Additional file 1:** **Table S1**. List of primers used for RAPD analysis | | |
| --- | --- | --- |
| No | Primer | Sequence (5’-3') |
| 1 | A-03 | AGT CAG CCA C |
| 2 | A-15 | TTC CGA ACC C |
| 3 | AA-19 | TGA GGC GTG T |
| 4 | AL-04 | ACA ACG GTC C |
| 5 | E-12 | TTA TCG CCC C |
| 6 | G-10 | CCG ATA TCC C |
| 7 | H-14 | ACC AGG TTG G |
| 8 | O-05 | CCC AGT CAC T |
| 9 | T-04 | GTC CTC AAC G |
| 10 | J-19 | GGA CAC CAC T |
| 11 | AA-01 | AGA CGG CTC C |
| 12 | AF-06 | CCG CAG TCT G |
| 13 | I-04 | CCG CCT AGT C |

PCR cycle conditions were: 1 cycle at 95℃ for 2 min, 45 cycles of 95℃ for 20 sec, 28.9℃ or 33.0℃ for 40 sec, 72℃ for 1 min with a final cycle of 5 min at 72℃. The primers described in the list are collected from the study of Biswas et al (2009).
